# A Retrospective Evaluation of Phenobarbital and Benzodiazepine Monotherapy and Combined Therapy in the Treatment of Alcohol Withdrawal Syndrome in a Canadian Emergency Department

**DOI:** 10.7759/cureus.109995

**Published:** 2026-05-31

**Authors:** David Jerome

**Affiliations:** 1 Department of Family Medicine, University of British Columbia, Vancouver, CAN

**Keywords:** alcohol addiction, alcohol dependence, alcohol withdrawal syndrome, benzodiazepines, chronic alcohol intake, phenobarbital therapy

## Abstract

Purpose

The first-line treatment for alcohol withdrawal syndrome (AWS) in the emergency department (ED) is benzodiazepines. Phenobarbital is used as an alternative first-line treatment for AWS in admitted patients. This retrospective cohort study assessed the safety and efficacy of phenobarbital and benzodiazepines as first-line treatments of AWS in a Canadian ED.

Methods

A retrospective chart review was performed of patients seen at a Canadian tertiary care ED from January 2022 until December 2023. Patients were included if they were treated in the ED for AWS with phenobarbital or benzodiazepines, either as monotherapy or as combined therapy. The primary outcome was the time from medication administration to disposition (discharge or admission). Secondary outcomes included ED length of stay, time to medication administration, admission rates, 48-hour ED return visits, and adverse events. Statistical comparisons were performed using ANOVA and chi-square testing with post hoc analyses.

Results

A total of 342 patient encounters were included. The majority of patients (n = 203) were treated with benzodiazepine monotherapy, and 97 patients were treated with phenobarbital monotherapy. There was no significant difference in the primary outcome between the phenobarbital and benzodiazepine monotherapy groups. Combined use of phenobarbital and benzodiazepines was associated with a longer time to disposition, potentially reflecting patients with more severe symptoms or treatment-resistant cases. Overall, adverse events occurred in 11% of patients, with no significant difference between phenobarbital and benzodiazepine monotherapy groups.

Conclusion

Patients treated with phenobarbital monotherapy for AWS in the ED had a similar time from medication administration to ED disposition when compared to patients treated with benzodiazepine monotherapy. Phenobarbital monotherapy may be an appropriate first-line therapy for AWS in the ED; however, prospective studies are required.

## Introduction

Alcohol use has a large impact on the health of Canadians and places a significant burden on Canadian emergency departments (EDs). In 2020, there were over 652,000 ED visits in Canada related to alcohol use [1]. A 2019 analysis showed that over the preceding decade, presentations to EDs in Ontario, Canada, for alcohol-related conditions increased 34% overall, and they increased 300% for women [2]. Among individuals with alcohol use disorder, approximately 50% of individuals will develop alcohol withdrawal syndrome (AWS) when their alcohol use is abruptly discontinued [3]. Patients with alcohol use disorder are therefore at risk of developing AWS whenever they present to the ED, whether for an alcohol-related condition or another indication. Medical and trauma patients experiencing AWS are at risk of increased morbidity and mortality, such as longer admissions to the intensive care unit (ICU) and higher rates of mechanical ventilation [4,5].

Benzodiazepines are broadly accepted as the first-line treatment for AWS in the ED [6]. Patients who drink heavily, however, are at risk of alcohol withdrawal that is resistant to benzodiazepines, thought to be due to chronic desensitization of the GABA (gamma-aminobutyric acid) receptor [7]. Phenobarbital is a barbiturate medication sometimes used as an alternate treatment for severe or benzodiazepine-resistant AWS [6]. It acts on GABA receptors at a different site than alcohol and benzodiazepines, and also acts on glutamate receptors. Phenobarbital is sometimes used as a first-line treatment for AWS in patients admitted to the ICU; however, its use in the ED is much less common [8]. A recent systematic review by the Guidelines for Reasonable and Appropriate Care in the Emergency Department (GRACE) Initiative concluded that phenobarbital may be an appropriate first-line alternative to benzodiazepines for the management of all cases of AWS treated in the ED [9]. The review was limited, however, by the low number and limited quality of the studies available for inclusion, and the authors called for more research on the topic. This study is a retrospective cohort study comparing the performance of phenobarbital and benzodiazepines as first-line treatments for AWS in a single Canadian tertiary ED.

## Materials and methods

Study design

A retrospective chart review was conducted of all patients who presented to the study site ED with AWS between January 2022 and December 2023. The study site is a tertiary care referral center in British Columbia, Canada, that sees an average of 82,000 patients annually in the ED. Patients were included if they received a Canadian Emergency Department Information System (CEDIS) presenting complaint code of 405 (seizure), 751 (substance misuse/intoxication), 752 (overdose ingestion), or 753 (substance withdrawal). Patients were excluded from the analysis if they were less than 18 years old, were not diagnosed with AWS during the ED visit, or did not receive either phenobarbital or a benzodiazepine during the ED visit.

A search of the electronic medical record (EMR) identified patients who met the inclusion criteria. A medical researcher blinded to the study purpose and hypothesis then completed a manual chart review and extracted clinical data using a standardized data abstraction template. The review included an assessment of all physician and nursing notes and the Medication Administration Record (MAR) from each patient encounter. A full list of the items listed in the abstraction template is available in the Appendices. Charts with missing information were excluded from analysis.

In order to assess the accuracy of the manually extracted study data, the author independently reviewed 50 randomly selected charts and noted discrepancies with the extracted dataset.

The Strengthening the Reporting of Observational Studies in Epidemiology (STROBE) guidelines for reporting observational research were followed during study design and execution [10]. The study received approval from the Interior Health Research Ethics Board (Study ID: 2024-25-033-H) and the University of British Columbia Research Ethics Board (Study ID: H24-01801).

Outcome measures

Outcomes were compared between three groups: patients who received phenobarbital only (PB group), patients who received benzodiazepines only (BZ group), and patients who received both phenobarbital and benzodiazepines during the same ED visit (PB+BZ group). The predetermined primary outcome was the time from the first administration of phenobarbital or benzodiazepine to ED disposition (either ED discharge or hospital admission). The study hypothesis was that phenobarbital would be associated with a lower time from medication administration to disposition.

Secondary outcomes included ED length of stay, time from ED arrival to study medication administration, total number of doses of study medication given, hospital admission rates, the rate of return visits to any ED within 48 hours of ED discharge, and the rate of adverse events associated with study medication administration. Adverse events were predefined as any mention in nursing or physician documentation of sedation or Glasgow Coma Scale (GCS) score less than 14, hypoxia, basic airway management (oxygen administration, manual opening of the airway, oral-pharyngeal airway insertion, nasal-pharyngeal airway insertion, application of artificial respirations), intubation, respiratory therapist consultation or assessment, or any other comments explicitly referencing a side effect from phenobarbital or benzodiazepine administration.

Data analysis

Descriptive statistics were calculated for primary and secondary outcomes. Continuous variables were assessed using one-way ANOVA. When statistical significance was identified, post hoc testing was performed using Tukey honestly significant difference (HSD) tests. Categorical variables were assessed with chi-square tests, and post hoc testing was performed using Bonferroni correction. All statistical analyses were completed using IBM SPSS Statistics version 25 (IBM Corp., Armonk, NY) [11].

## Results

**Figure 1:**
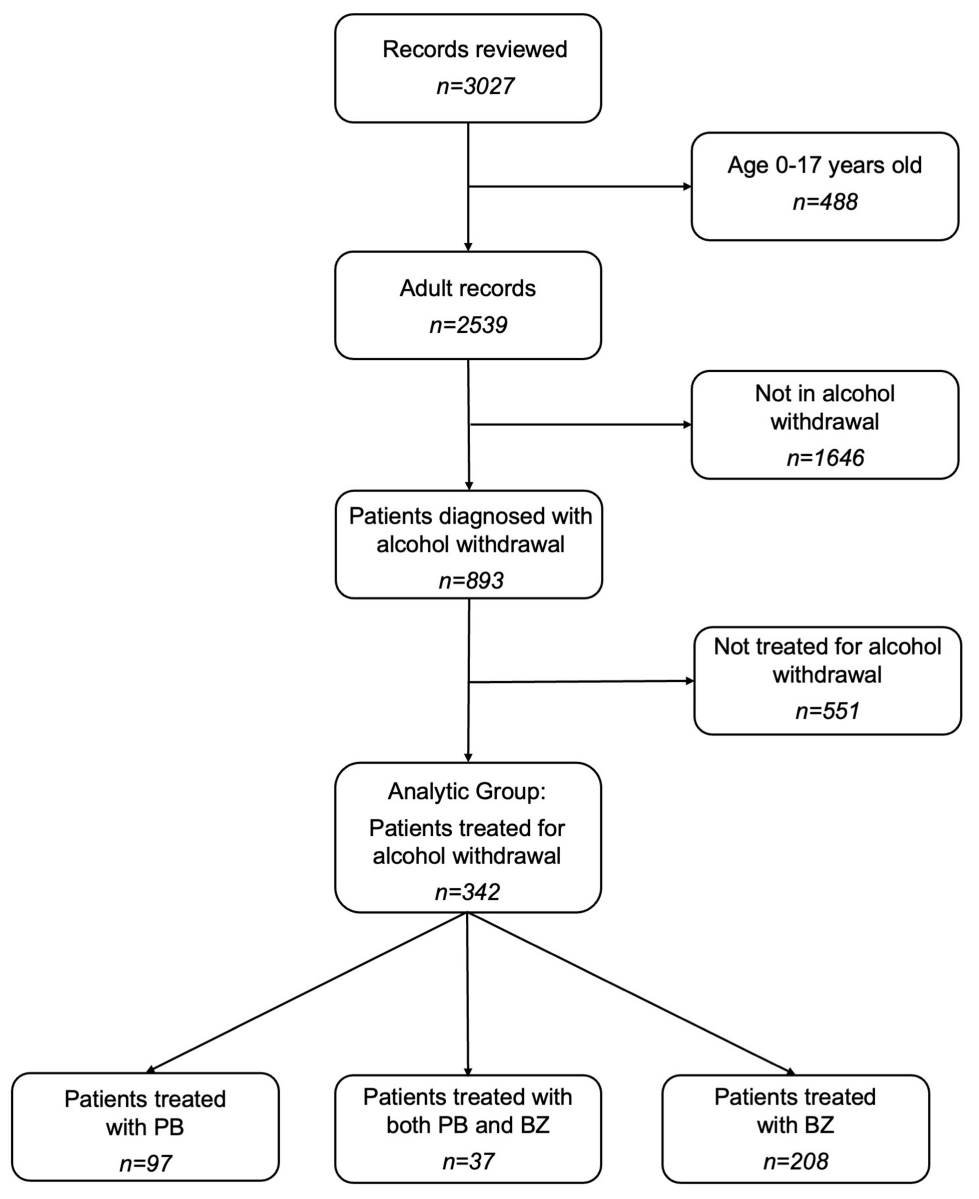
Flow diagram for study participants. PB: phenobarbital; BZ: benzodiazepines.

There were 3027 patient encounters associated with one of the four flagged CEDIS codes during the study period. After application of the exclusion criteria, 342 patient encounters remained in the analytic sample (Figure 1). The majority of patients (203) were in the BZ group. There were 551 patients diagnosed with AWS who did not receive treatment in the ED. The most common reasons documented in the chart for not treating AWS included the following: the symptoms of AWS were mild, the patient left against medical advice before treatment could be initiated, or the patient was dispositioned before treatment could be initiated.

Patient characteristics are presented in Table 1. Females represent 30% of the study group. The mean highest Clinical Institute Withdrawal Assessment for Alcohol Scale (CIWA-Ar) was 18.5, and 49% of the group had a history of alcohol withdrawal seizures. The three study groups were similar across all baseline characteristics with the exception of mean initial systolic blood pressure, which was significantly lower in the BZ group (p = 0.01). Patients in the PB group received a mean cumulative dose of 579 mg.

**Table 1:** Patient characteristics. BZ: benzodiazepine; CTAS: Canadian Triage and Acuity Scale; CIWA-Ar: Clinical Institute Withdrawal Assessment for Alcohol – Revised scale; ED: emergency department; NA: not applicable; PB: phenobarbital; SBP: systolic blood pressure; SD: standard deviation. * indicates statistical significance.

Characteristic	PB only (n = 97)	BZ only (n = 208)	Both (PB + BZ) (n = 37)	p-value	F-value	x^2^ value
Demographics						
Female	30%	31%	22%	0.50	NA	χ² (2, N = 342) = 1.39
Age in years	46	43	42	0.15	F (2, 339) = 1.91	NA
History of alcohol withdrawal seizure	49%	47%	62%	0.22	NA	χ² (2, N = 342) = 3.04
Clinical presentation						
CTAS level	2.6	2.6	2.5	0.5	F (2, 339) = 0.69	NA
Initial CIWA-Ar	13.8	14.3	13.9	0.94	F (2, 209) = 0.07	NA
Highest CIWA-Ar	19.7	17.7	19.5	0.36	F (2, 209) = 1.03	NA
Initial pulse	105	101	105	0.21	F (2, 339) = 1.56	NA
Initial SBP	145	137	142	0.01*	F (2, 339) = 5.22	NA

**Figure 2:**
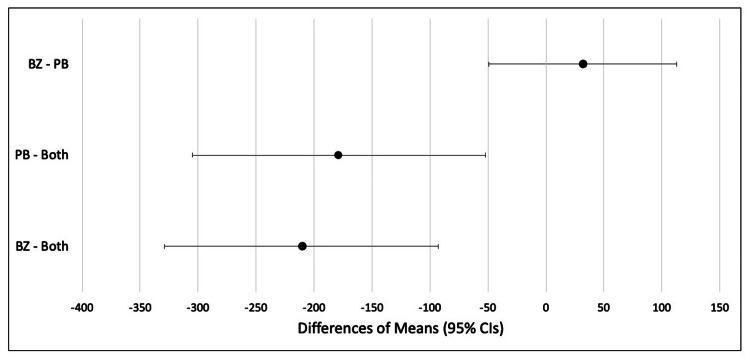
Tukey post hoc analysis of time from first study medication administration to ED disposition. BZ: benzodiazepines; PB: phenobarbital.

The difference in the primary outcome of time from study medication administration to ED disposition was statistically significant across the three groups (p = 0.00), with the PB+BZ group having the highest mean time at 449 minutes (Table 2). Post hoc analysis showed there were significant differences between the PB+BZ/PB (p = 0.00) and PB+BZ/BZ groups (p = 0.00), but not between the PB/BZ groups (p = 0.63) (Figure 2).

**Table 2:** Primary and secondary outcomes. BZ: benzodiazepine; ED: emergency department; NA: not applicable; PB: phenobarbital; SD: standard deviation. * indicates statistical significance.

Characteristic	PB only	BZ only	Both	p-value	F-value	χ² value
Primary outcome						
Time from first medication administration to discharge/admission - minutes	270	240	449	0.00*	F (2, 339) = 8.94	NA
Secondary outcomes						
Time from ED arrival to first medication administration - minutes	158	169	124	0.31	F (2, 339) = 1.19	NA
ED length of stay - minutes	428	407	573	0.03*	F (2, 339) = 3.67	NA
Total number of doses of study medication	1.6	1.7	3.3	0.00*	F (2, 339) = 20.90	NA
Received non-study medications during ED stay	78%	75%	97%	0.00*	NA	χ² (2, N = 342) = 9.53
Experienced an adverse event	8%	10%	24%	0.02*	NA	χ² (2, N = 342) = 7.56
Held in ED for observation	14%	12%	16%	0.72	NA	χ² (2, N = 342) = 0.65
Admitted as an in-patient	44%	38%	59%	0.04*	NA	χ² (2, N = 342) = 6.22
Return presentation to the ED in the next 48 hours	6%	11%	11%	0.39	NA	χ² (2, N = 342) = 1.87

The difference in ED length of stay was statistically significant across the three groups, with post hoc analysis showing a significant difference between the PB+BZ/BZ groups (p = 0.02) but not the PB+BZ/PB groups (p = 0.07) or the PB/BZ groups (p = 0.09). There was no significant difference, however, in time from ED arrival to first medication administration, or in the rate of ED return in 48 hours (Table 2). There was a statistically significant difference in the rates of inpatient admission, with the PB+BZ group having the highest rate at 59% (p = 0.04).

A total of 38 patients experienced adverse events, representing 11% of patients. Nearly a quarter of these were in the PB+BZ group, though this group only represented 11% of the analytic sample. There was no significant difference in the rate of adverse events between the PB and BZ groups (p = 0.61). The most common adverse event patients experienced was a basic airway intervention (n = 19), representing half of the documented adverse events (Table 3). This was closely followed by GCS less than 14 (n = 18).

**Table 3:** Types and rates of adverse events. AE: adverse event; GCS: Glasgow Coma Scale. * Some patients experienced more than one adverse event, so the sum of the rates adds up to more than 100%.

Type of AE	Number of AE (n = 51)	Rate per patient with AEs (n = 38)*	Rate per total patients (n = 342)
Basic airway intervention	19	50.0%	5.5%
Intubation	3	7.9%	0.9%
Seizure	7	18.4%	2.0%
GCS < 14	18	47.4%	5.3%
Other	4	10.5%	1.1%

Of the 50 charts reviewed to assess the fidelity of the dataset, one record had a discrepancy in the total number of doses and the cumulative administered dose of the study medication.

## Discussion

In this large retrospective study of patients treated for AWS in a Canadian tertiary hospital, patients treated with phenobarbital and benzodiazepine monotherapy did not have significant differences in the time from first medication administration to disposition. The two groups experienced similar rates of adverse events. There was also a trend toward lower rates of return presentation to the ED in patients who received phenobarbital (6%) compared to benzodiazepines (11%), but this did not reach statistical significance. Patients who received both study medications, however, had a statistically longer time from medication administration to disposition, were more likely to be admitted to the hospital, and experienced a higher rate of adverse events. These observations could be due to an interaction between the medications, or these patients may have represented more complex cases of AWS resistant to the initial treatment provided. Prospective trials are required to further assess the effectiveness and safety of monotherapy and combined therapy with these medications in the treatment of AWS.

A growing body of evidence suggests that phenobarbital performs equally to benzodiazepines when used to manage AWS in patients admitted to the ICU [8,12,13] or general inpatient units [3,14]. Studies assessing this question in ED patients have suggested similar findings, but have been mostly based in the United States and limited by small sample sizes [15-18]. The findings and large sample size in the current study strengthen the body of ED-related literature on the role of phenobarbital in the management of AWS. This study, together with previous studies performed in the ED, suggests that phenobarbital may be non-inferior to benzodiazepines as a first-line management for AWS in the ED.

There is a lack of evidence informing the optimal dosing of phenobarbital in the treatment of AWS. A common low-dose approach involves administering an initial IV dose of 260 mg based on ideal body weight (IBW), followed by titration with 130 mg IV as required [15,19]. An alternate high-dose strategy has been described, in which patients are front-loaded with an initial dose of 10 mg/kg IBW [3,17,18]. The only study published to date that directly compared the low-dose and high-dose strategies was a retrospective single-site study that enrolled 176 patients treated for AWS while admitted to the hospital [3]. Patients who received the low dose of 6 mg/kg IBW had a longer hospital length of stay. The study reported no significant difference, however, in the rates of adverse events between the two study groups. The dosing of phenobarbital was not protocolized at the study site during the current study. The mean total dose of phenobarbital patients received was 579 mg in the PB group and 462 mg in the PB+BZ group. Patient weights were not regularly recorded; however, these doses are likely well under 10 mg/kg, as a 70 kg adult would receive a 700 mg loading dose. It is possible that patients treated with an initial dose of 10 mg/kg IBW of phenobarbital may have improved outcomes, and that patients treated with phenobarbital in the current study were therefore underdosed.

A history of alcohol withdrawal seizure and a CIWA-Ar score >19 are both risk factors for severe AWS [6]. In the current study, 49% of patients had a history of alcohol withdrawal seizure, and the average highest CIWA-Ar score was 18.5, indicating that a high proportion of patients presenting to Canadian EDs are experiencing severe AWS. Given that the rate of ED presentations for alcohol-related indications is increasing, the volume of severe AWS presentations is likely to rise as well [1,2]. Continuing to investigate alternative medications and dosing strategies will help optimize the management of this growing population of patients presenting with AWS.

Strengths and limitations

The high degree of accuracy identified during the review of the study dataset reflects strong data collection methods. The primary outcome of time from medication administration to ED disposition was selected because it was considered to be patient-oriented and a potential surrogate for time to symptom resolution, since symptom resolution is usually the threshold for discharging patients with AWS from the ED. Measuring symptom resolution directly was not possible, as a preliminary review of the EMR indicated that many records did not have CIWA-Ar scores recorded through to the end of the ED visit. This is consistent with other studies investigating AWS and likely represents the effect of increased pressure on nursing staff in the context of ED crowding and trends toward higher patient acuity [19,20].

As a retrospective analysis, it is not possible to identify or assess the effect of confounding variables, such as individual physician practice patterns. The use of CEDIS codes to include enrolled patients means that some patients may have been missed. As CEDIS codes are applied during the triage process, patients who presented to the ED with another medical problem and then developed alcohol withdrawal while in the department would not be represented in this dataset. The EMR used at the study site is linked to all other hospitals in the regional health authority. The site is centrally located within the health authority’s geographic area of 215,000 square kilometers; therefore, it is likely that patients who had a repeat visit to an ED within 48 hours of their index visit did so within the health authority and would have been identified. It is possible, however, that some patients were seen in follow-up outside the health authority, in which case the visit would have been missed.

Research implications

Future research efforts should be aimed at running large prospective randomized trials evaluating phenobarbital as a first-line treatment for AWS in the ED. Studies should specifically assess the performance and safety of phenobarbital dosed at 10 mg/kg IBW. It would also be valuable to assess alternate routes of phenobarbital administration for clinical efficacy (intramuscular, oral) for use in milder cases of AWS and in outpatient settings.

## Conclusions

In this retrospective cohort study conducted at a Canadian tertiary ED, phenobarbital and benzodiazepine monotherapy demonstrated comparable performance with respect to time from medication administration to disposition, with similar rates of adverse events. These findings support the growing body of evidence that phenobarbital is a reasonable first-line alternative to benzodiazepines for the management of alcohol withdrawal syndrome in the ED setting. Patients who received both study medications experienced longer times to disposition, higher admission rates, and more adverse events, although this may reflect greater illness severity rather than a direct medication effect.

Further prospective, randomized studies are warranted to better define the role of phenobarbital in ED protocols, including optimal dosing strategies and patient selection. In particular, evaluation of higher weight-based dosing regimens and alternative routes of administration may help clarify its utility across the spectrum of alcohol withdrawal severity.

## Appendices

Metrics from data and analytics

• Account Number

• Unit Number (Terminal Digit Format)

• Patient's Legal Name

• Health Care Number

• Patient's Gender

• Patient's Age at Admission

• Patient's Place of Residence (urban/rural by the first 3 letters of the postal code)

• ED Presenting Complaint Code - Canadian Emergency Department Information System (CEDIS)

• ED Presenting Complaint Name - CEDIS

• ED Arrival Date - Day Name

• ED Arrival Date - Month Name

• ED Arrival Time - Hourly Group

• Was Admitted as an Inpatient?

• ED Visit Start Date and Time to Admit Date and Time

• Admit Date and Time to ED Departure Date and Time

• Valid Admit Date and Time to ED Departure Date and Time

• First In-Patient Location

• ED Departure Disposition

• ED Departure Disposition Description

• ED Length of Stay (Minutes)

• Valid ED Length of Stay (LOS)

• Canadian Triage and Acuity Scale (CTAS) Level

• Revisit Within 72 Hours? - Manual Calculation

• Revisit Date

• Nursing Unit Location Name

• Nursing Unit Start Date and Time

• Nursing Unit End Date and Time

• ICU LOS

• Admit Date and Time

• Discharge Date and Time

• Length of Stay

Metrics from manual chart review

• Alcohol Use Withdrawal (Y/N)?

• History of Alcohol (EtOH) Withdrawal Seizure

• Initial Pulse

• Initial Systolic Blood Pressure

• IV Start

• Start Time of Drug Administration

• Time from ED start date/time to first benzo/barb administered

• Route of Administration

• Number of Doses Administered

• Clinical Decision Unit (CDU) Observation in the Emergency Room (ER)

• Initial CIWA score

• Highest CIWA Score

• Other Medications Given (Y/N)?

• Other Medication Given 1

• Other Medication Given 2

• Other Medication Given 3

• Was a Discharge Prescription Given (Y/N)?

• What Medication Was the Discharge Prescription For?

• Co-morbid Health Condition 1

• Co-morbid Health Condition 2

• Co-morbid Health Condition 3

• Co-morbid Health Condition 4

• Co-morbid Health Condition 5
